# Comparison of the Efficacy of Transdermal Buprenorphine Versus Ketoprofen Patches for Post-operative Analgesia in Total Knee Arthroplasty: A Randomised Controlled Trial

**DOI:** 10.7759/cureus.72382

**Published:** 2024-10-25

**Authors:** Tushar Nayak, Mukund Madhav Ojha, Mohd Akhtar Ansari, Sandeep Sehrawat, Vivek Shankar, Shailendra Kumar, Vijay Kumar

**Affiliations:** 1 Orthopaedics, All India Institute of Medical Sciences, New Delhi, New Delhi, IND; 2 Orthopaedics, All India Institute of Medical Sciences, Bathinda, Bathinda, IND; 3 Anaesthesiology, All India Institute of Medical Sciences, New Delhi, New Delhi, IND

**Keywords:** buprenorphine, ketoprofen, knee arthroplasty, postoperative pain, transdermal patch

## Abstract

Introduction

The utilisation of transdermal patches containing buprenorphine (BTP) and ketoprofen (KTP) has been widely documented for post-operative pain management. However, to date, no single study has comprehensively evaluated the efficacy of KTP and BTP specifically in managing post-operative pain following arthroplasty procedures.

Methods

A total of 100 consecutive patients undergoing primary total knee replacement between August 2022 and January 2023 were randomly assigned into two groups (Group KTP and BTP) using computer-generated numbers. Patients in Group BTP received a buprenorphine patch (10 µg/h) 24 hours before surgery, replaced on the seventh day and sustained for 14 days. Group KTP received a ketoprofen patch (20 mg) on the day of surgery, replaced daily for 14 days. Pain intensity was assessed using the Visual Analog Scale (VAS) score pre-operatively and post-operatively. Clinical outcomes included VAS scores at baseline, on post-operative days (POD) 2, 5, and 14, both at rest and during activity, need for rescue analgesia, adverse events, duration of hospital stay, post-operative range of motion (ROM), and patient satisfaction.

Results

Results revealed significantly lower VAS scores at rest in the BTP group for the initial five days, with similar trends observed for VAS scores during activity. No significant differences were found in VAS scores at day 14. There was an increased need for rescue analgesia in the KTP group compared to the BTP group. Post-operative ROM was consistently higher in the BTP group, although not statistically significant. Patient satisfaction scores favoured the BTP group throughout admission, with no remarkable adverse effects in either group.

Conclusion

In conclusion, BTP patches demonstrated enhanced efficacy in managing post-operative pain compared to KTP, without exacerbating side effects in the early stages following total knee arthroplasty. BTP can be definitively considered as an adjuvant in post-operative total knee arthroplasty analgesia.

## Introduction

Post-operative pain following total knee arthroplasty (TKA) is one of the foremost concerns for orthopaedic surgeons and anaesthetists alike, potentially influencing the procedure’s overall results [1,2]. Some modalities like femoral nerve block endorsed for managing post-operative pain are invasive and interfere with immediate post-operative mobilisation [3-5]. Unlike oral and parenteral administration, transdermal drug delivery systems (TDDS) elute a sustained drug concentration and reduce side effects compared with other routes. As a result of their convenience and non-invasiveness, their use has been preferred in clinical settings [6].

Buprenorphine (semi-synthetic opioid) is consistently used to control acute and chronic pain [7,8]. Its greater half-life and increased affinity to the ‘m’ receptors make it longer acting and more effective than morphine [5]. Its molecular composition allows it to be used in various formulations, including transdermal preparations. As a transdermal patch (TDP), buprenorphine is integrated into an adhesive polymer matrix, from which there is sustained release into the plasma for up to seven days. Buprenorphine transdermal patch (BTP) is now also increasingly used for pain relief in orthopaedic surgeries [9,10]. With well-established results for chronic pain, the data regarding the efficacy and safety of BTP in post-operative pain is limited [11]. The results of a few publications are encouraging for its use in acute pain management following arthroplasty procedures [6-8].

Ketoprofen, (propionic acid class of non-steroidal anti-inflammatory drugs (NSAIDs)) in various preparations, has been used as an effective analgesic in post-operative pain [12-14]. Ketoprofen transdermal patches (KTPs) are proven to be more successful than placebos in both traumatic and somatic pain [15,16]. The superior efficacy of transdermal ketoprofen to transdermal diclofenac in post-operative lower limb orthopaedic surgeries is also well reported [17,18]. However, there is no single study that has compared the efficacy of KTP and BTP in the management of post-operative pain following arthroplasty procedures.

Therefore, this RCT is designed to compare the effectiveness and safety of the BTP and KTP for post-operative analgesia following TKA. The hypothesis is that patients receiving the BTP would have a better post-operative analgesia and decreased need for rescue analgesia as compared to patients on a KTP.

## Materials and methods

Patients undergoing primary, total knee replacement, between August 2022 and January 2023, were included in the randomized controlled trial. This study was approved by the Institutional Ethics Committee (IEC-653/05.08.2022, RP-15/2022), and the trial was prospectively registered with the Clinical Trial Registry, India (CTRI/2022/12/048057). This trial was performed in accordance with the principles of the Declaration of Helsinki (Edinburgh 2000 version).

Based on the previous study published, sample size was calculated. The estimated sample size for a two-sample comparison of means is as follows: Test Ho: m1 = m2, where m1 is the mean in population 1, and m2 is the mean in population 2. The assumptions are as follows: alpha = 0.0500 (two-sided), power = 0.9000, m1 = 36, m2 = 32, sd1 = 3, sd2 = 3, and n2/n1 = 1.00. The estimated required sample sizes are as follows: n1 = 36 and n2 = 36.

Taking into account for lost to follow-up/drop-out rate of 20%, we decided to take 30 patients in each group. As we exceeded the required sample, we continued the trial till n = 50 in each group.

The analgesia protocol was explained, and informed consent was acquired in all patients. All of the patients were pre-operatively randomized into two groups (BTP and KTP) by computer-generated numbers. Tables 1, 2 represent the inclusion and exclusion criteria.

**Table 1:** Inclusion Criteria ASA: American Society of Anesthesiologist.

Inclusion Criteria
Patients aged between 18 and 85 years, scheduled to undergo a total knee arthroplasty
ASA grade I-III
No intra-articular procedures within the previous three months
No previous knee or hip procedures

**Table 2:** Exclusion Criteria

Exclusion Criteria
Allergies to opioids or NSAIDs
Revision surgeries
Subjects who will refuse enrolment or later request removal from the study
Patients with contraindications for regional anaesthesia
Pain and disability at any other site

All patients received spinal anaesthesia without any additional regional blocks. TKA was performed through the traditional anterior medial parapatellar approach using a cemented cruciate retaining or posterior stabilised system fixed without patellar replacement. Torniquet was inflated prior to cementing and deflated once the cement was set. We recorded the duration of surgery, and the total intra-operative blood loss was measured aptly after weighing sponges and subtracting lavage fluid from blood present in suction bottle. All patients received periarticular local anaesthetic infiltration. No drain was placed.

Patients in the BTP group received a BTP (10 µg/h) applied on a hairless aspect of chest or arm 12 hours prior to surgery. The patch was changed on the seventh day and was continued for 14 days. Similarly, the patients in the KTP group received a KTP (20 mg) on the hairless aspect of the chest or arm 12 hours before surgery, and the patch was changed daily for 14 days.

Post-operative analgesia consisted of Inj. Paracetamol 1 gm TDS and Inj. Diclofenac 50 mg TDS for two days and then shifted to oral formulations (tab celecoxib 200mg BD) for the next three days. Cryotherapy for half an hour QID was applied at the operated site for five days post-operatively. On complaints of pain at rest or activity of >4 on the VAS scale, Inj. Tramadol 50 mg was given as a rescue analgesic. On patients experiencing nausea or vomiting, Inj. Ondansetron 4mg was administered. All patients received Inj. Cefuroxime 3 gm daily for five days in divided dosage. Tab. Aspirin (75 mg/day) was administered orally from postoperative day (POD) 1 to 30. All patients were mobilised and permitted full-weight-bearing on POD 1.

Intensity of pain was measured using the VAS score (range: 0 to 10) both prior to surgery and post-operatively. VAS at rest was obtained at two, six, 12, 24, and 48 hours and on the third, fifth, and 14th day, and VAS at activity was obtained at 24 hours and 48 hours and on the third, fifth, and 14th day following surgery. Clinical outcome was measured using the VAS as a baseline at pre-operative evaluation and on POD 2, 5, and 14. Need for rescue analgesia (IV Tramadol 50 mg stat dose) was noted in each group. Adverse events, duration of hospital stay, and patient satisfaction were noted. Patient satisfaction scores were measured on a scale of 1 to 5 (1 = least satisfied, 5 = most satisfied).

Study data was presented as means ± SD. All statistical tests were evaluated at the 5% level of significance. The VAS score and rescue analgesia requirement were assessed by the independent T test, and dichotomous variables, such as the incidences of adverse effects (AEs), were compared by the Chi-squared test or Fisher’s exact test. All statistical analyses were performed using SPSS software (version 20.0; SPSS, Chicago, IL, USA).

## Results

**Figure 1:**
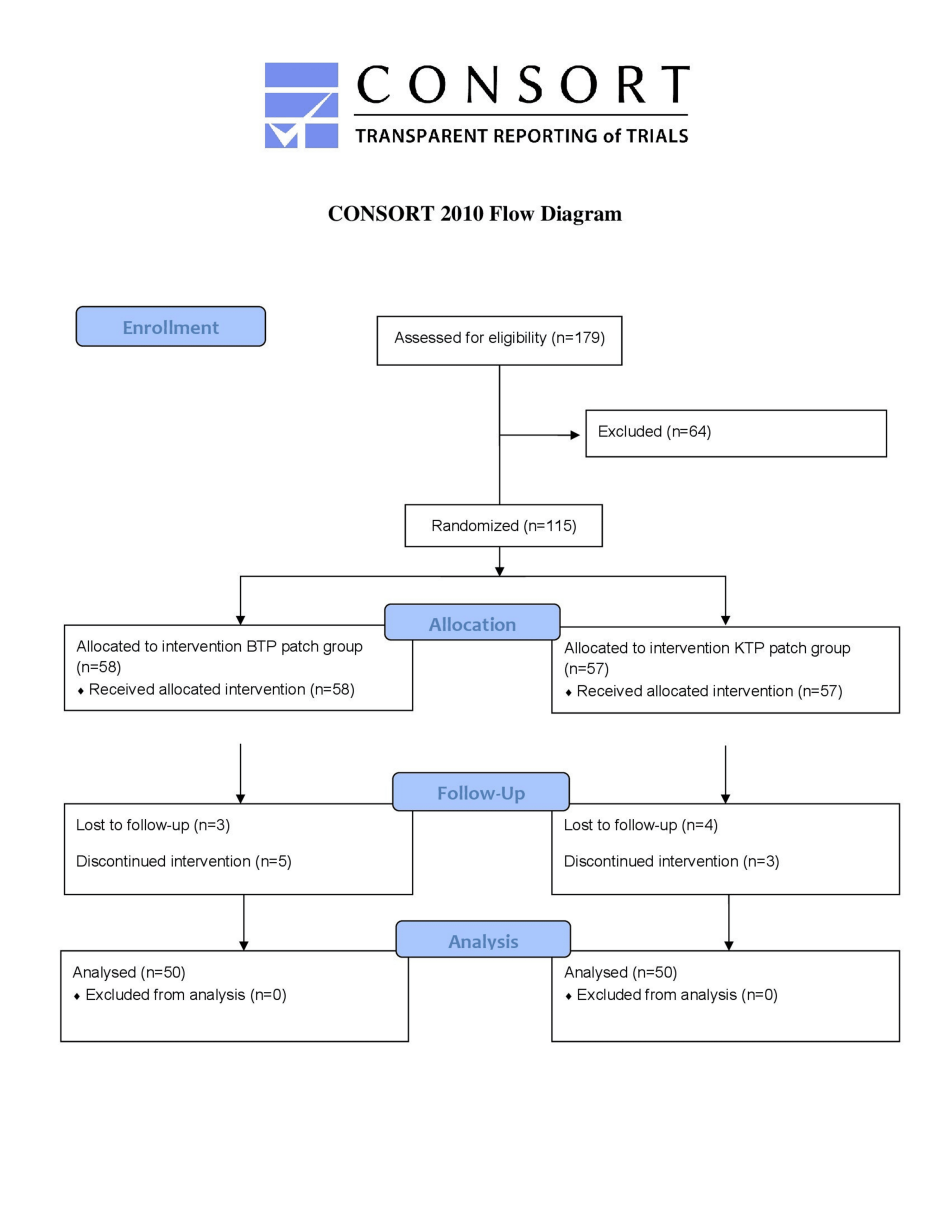
CONSORT Flow Diagram CONSORT: Consolidated Standards of Reporting Trials, BTP: buprenorphine transdermal patch, KTP: ketoprofen transdermal patch.

Figure 1 shows the CONSORT flow diagram. The two groups had no significant differences in demographics and clinical and operative characteristics (Table 3). VAS scores at rest were significantly lower in the BTP group for the initial five days, p = 0.0027, p = 0.0001, and p = 0.0050 on day 1, day 2, and day 3, respectively (Table 4). VAS scores on activity were also found to be significantly lower in the BTP group for the initial five days (Table 5). There was no significant difference in VAS scores on day 14 p=0.5437 at rest and p=0.3170 at activity. Range of motion (ROM) in the BTP group at all days post-operatively was higher as compared to that in the KTP group, but values were not statistically significant (Table 6). On all days of admission, the patient satisfaction scores were statistically higher in the BTP group at 12 hours (p = 0.0002), on day 2 (p = 0.0012), and on day 3 (p=0.0000) as compared to the KTP group (Table 7). Twenty patients in the KTP group needed rescue analgesia as compared to eight in the BTP group (Table 8). Patient satisfaction scores were comparable in both BTP and KTP group on day 14 (p = 1.0000).

**Table 3:** Demographic and Operative Characteristics BTP: buprenorphine transdermal patch, KTP: ketoprofen transdermal patch, ASA: American Society of Anesthesiologist.

		BTP (n = 50)		KTP (n = 50)	p value
Age (years)		62.52±4.385		63.24±4.880	0.8002
Gender		Male:	19	17	
		Female:	31	33	
ASA grade	I	10		13	
	II	36		34	
	III	4		3	
Alignment		Varus	46	47	
		Valgus	4	3	
BMI		23.7±1.864		23.768±1.680	0.8034
Blood loss (ml)		266.04±45.071		269.68±45.962	0.5289
Operative time (minutes)		69.3±12.435		64.9±12.673	1.0000
Length of hospital stay (days)		6.38±1.550		6.6±1.653	0.4476

**Table 4:** VAS Score (at Rest) VAS: Visual Analogue Scale, BTP: buprenorphine transdermal patch, KTP: ketoprofen transdermal patch.

	Pre-operative	2 hr	4 hr	8 hr	12 hr	24 hr	48 hr	72 hr	5th day	14th day
BTP	5.84±1.075	1.32±0.512	1.7±0.614	1.8±0.755	1.6±0.606	2.3±0.707	1.92±0.944	1.4±0.699	1.32±0.586	1.22±0.464
KTP	5.92±1.121	1.74±0.750	2.28±0.881	2.36±0.898	2.28±1.030	2.94±1.235	2.42±1.051	2.04±0.924	1.70±0.952	1.32±0.620
P value	0.7277	0.0037	0.0004	0.0011	0.0004	0.0027	0.0050	0.0001	0.0366	0.5437

**Table 5:** VAS Score (at Activity) VAS: Visual Analogue Scale, BTP: buprenorphine transdermal patch, KTP: ketoprofen transdermal patch.

	Pre-operative	24 hr	48 hr	72 hr	5th day	14th day
BTP	7.5±1.147	2.8±3.028	2.78±0.840	2.32±0.913	1.88±0.718	1.46±0.503
KTP	7.4±1.061	3.44±2.935	3.54±0.734	2.9±0.839	2.26±0.828	1.58±0.498
p value	0.4275	0.0000	0.0000	0.0009	0.0108	0.3170

**Table 6:** ROM Outcome in 2 Groups ROM: range of motion, POD: post-operative day, BTP: buprenorphine transdermal patch, KTP: ketoprofen transdermal patch.

	Pre-operative	ROM POD2	ROM POD5	ROM POD14
BTP	82.02±11.024	54.5±8.406	92.4±15.722	115.66±11.307
KTP	81.84±7.635	53±6.851	88±12.453	115.06±12.194
p value	0.8376	0.1118	0.0759	0.7746

**Table 7:** Patient Satisfaction Score BTP: buprenorphine transdermal patch, KTP: ketoprofen transdermal patch.

	6 hr	12 hr	24 hr	48 hr	72 hr	5th day	14th day
BTP	4.4±0.642	4.16±0.765	3.9±0.863	4.58±0.758	4.8±0.404	4.82±0.388	4.72±0.453
KTP	4.08±0.812	3.58±0.672	3.76±0.846	3.98±0.958	4.26±0.599	4.64±0.484	4.74±0.443
p value	0.0453	0.0002	0.4331	0.0012	0.0000	0.0705	1.0000

**Table 8:** Rescue Analgesia Required Within 72 Hours BTP: buprenorphine transdermal patch, KTP: ketoprofen transdermal patch.

Group	<72 hr
BTP	8
KTP	20

The incidence of adverse effects was not statistically significant between the two groups (Table 9). However, the incidence of post-operative nausea vomiting (PONV), urinary retention, and constipation was higher in the BTP group. No patients had respiratory depression. Two patients in the BTP group and five patients in the KTP group had localised erythema around the patch site. Wound soakage and superficial stich abscess were the skin complications, which healed on conservative management and antibiotics and were unrelated to the patch. Two patients, one in either group, had deep vein thrombosis (DVT) in post-operative period and were promptly managed with low molecular heparin after consultation with the medicine department.

**Table 9:** Adverse Events BTP: buprenorphine transdermal patch, KTP: ketoprofen transdermal patch, DVT: deep vein thrombosis.

	BTP	KTP
Nausea	8	6
Vomiting	7	5
Dizziness	None	None
Headache	6	8
Respiratory depression	None	none
Urine retention	4	3
DVT	1	1
Incisional complications	3	4
Dyspepsia	2	3

## Discussion

This study compared the analgesic efficacies of a transdermal opioid (10 µg BTP) with a transdermal NSAID (20 mg KTP) in the early post-operative period in patients undergoing a primary TKA. The patients in the BTP group had lower pain scores, reduced need for rescue analgesia, and a higher patient satisfaction score than the KTP group. There were no significant differences in the adverse effects.

Inadequate post-operative analgesia often leads to a delay in physiotherapy, and prolonged hospital stays, and may even adversely affect the outcome of surgery. Early mobilisation may occasionally be delayed by the use of epidural analgesia and continuous blocks during the post-operative phase. TDDS are safe, easy to use, non-invasive, and a reliable route for drug administration. Due to the slow and sustained release of drugs, sudden increases in plasma drug levels are avoided, reducing the incidence of adverse effects associated with drugs. By maintaining continuous, sustained plasma levels, the incidence of break-through pain and requirement for rescue analgesia are reduced.

BTPs are commercially available as 5, 10, and 20 mg patches eluting the drug at 5, 10, and 20 μg/h, respectively. Analgesic efficacy is directly proportional to a dose up to 17.5 to 20 µg/hr, with no further analgesic benefits at higher doses [19,20]. The drug-related side effects also increase proportionally; however, there are no reported severe/life-threatening adverse effects up to a dose of 52.5 µg/h [18,19]. As the usual dose of ketoprofen varies, the dose of the commercially available 20 mg ketoprofen patch was used in the study. In order to reach peak plasma concentration, BTP was applied 12 hours prior to surgery [21,22] and was changed every seven days, while KTP was applied on the morning of surgery and was changed every 24 hours as per manufacturer recommendations. KTP 100 mg patch has only 10% systemic bioavailability compared to ketoprofen 100 mg orally. However, BTP's low molecular weight, high lipophilicity, and potency make it ideal for transdermal formulations [23].

The safety and efficacy of pre-operative BTP in the elderly are well reported by Conaghan et al. [24] in proximal femoral fractures and osteoarthritis of large joints, respectively. Sustained analgesia with negligible side effects after hallux-valgus correction was observed by Pergolizzi and Raffa [8], and Fujita et al. [4] suggested that the efficacy of BTP is similar to oral tramadol following in spinal fusion. Privitera and Guzzetta [25] and Pergolizzi et al. [7] in their distinct descriptive analyses using BTP for patients undergoing shoulder, femur, and lumbar spine surgeries concluded that BTP is safe and effective for post-operative analgesia with greater satisfaction scores. Xu et al. [6] stated that BTP provides effective pain relief and reduces rescue morphine with no increase in adverse effects as compared to oral celecoxib in the initial periods of post-operative analgesia following TKA.

Every method of NSAID use lowers the need for opioids and hospital stays [26]. In addition, ketoprofen, with its low molecular weight of 260 Da, has the fastest and highest cutaneous permeability of any NSAID and is found to be more effective when applied topically than other NSAIDs like diclofenac [27]. The efficacy of transdermal ketoprofen following tooth extractions, sports injuries, and post-operative analgesia in lower limb orthopaedic surgeries is well reported, and Kawai et al. [16] reported its superiority over transdermal diclofenac in hip surgeries.

In the current study, post-operative VAS scores were significantly lower in the BTP group both at rest and during mobilisation on all points except on the 14th post-operative day. Also, the need for rescue analgesia was significantly lower in the BTP group. Although the mean post-operative ROM on POD 1, 2, 3, 5, and 14 was higher in the BTP but not statistically significant, indicating that buprenorphine is more useful than ketoprofen in immediate post-operative mobilisation. Maximum demand (32%) of rescue analgesia was observed at 24 hours post-surgery. This corresponds to the time when patients are mobilised full wight bearing and are instructed to perform active quadriceps strengthening exercises. This also corresponds to the weaning off the intra-articular local anaesthetic preparations. The overall need for rescue analgesia was higher in the KTP group as compared to the BTP group.

BTP was associated with higher rates of PONV, urine retention, and constipation compared to KTP, but these differences were not statistically significant. The side effects are largely similar in incidence, as established by post-marketing surveillance in both drugs, unlike the higher incidences when buprenorphine was used for cancer pain as reported by Tassinari et al. [28]. Previous studies have reported respiratory depression and dizziness were associated with buprenorphine; however, no such events were observed in the 10-µg patch used in our study. In the current study, no severe adverse effects were observed as to withdraw the application of either patch. Cutaneous side effects mainly included erythema, rash, and itching which were similar in both groups and also similar to reported placebo trails. There was no significant incidence of dyspepsia in the KTP group, and the overall incidence was less than 12% of the reported incidence of oral NSAIDs [29].

There are few limitations to this study. There was blinding of patients and analysing observers during treatment and data analysis. TDDS are slow in onset and have a sustained effect, making it inferior for immediate pain relief, where patient-controlled analgesia performs better. The dosage of the two drugs were chosen with reference to the manufactures’ recommendations and their efficacies in previously published articles. However, it is unclear if dosage of the comparative drugs used were equipotent to each other. Also, this study focused on the use of rescue analgesia and the use of intermittent multi-modal analgesia for post-operative period. As the pain is a subjective feeling and tolerance to pain varies from patient to patient, VAS score as criteria for providing rescue analgesia is not ideal. The regression of spinal anaesthesia in the initial post-operative period was not documented. Finally, we added a local anaesthetic cocktail in all outpatients which could form a bias for the efficacy of the initial analgesia of both the agents.

## Conclusions

The study indicates that BTP patches provide more effective pain relief and reduce the need for rescue analgesia as compared to KTP. Both the patches do not increase the side effects during the early post-operative stage following TKA.
